# Nucleolar Relocalization of RBM14 by Influenza A Virus NS1 Protein

**DOI:** 10.1128/mSphereDirect.00549-18

**Published:** 2018-11-14

**Authors:** Grant Beyleveld, Daniel J. Chin, Elena Moreno Del Olmo, Jade Carter, Isabel Najera, Cristian Cillóniz, Megan L. Shaw

**Affiliations:** a Department of Microbiology, Icahn School of Medicine at Mount Sinai, New York, New York, USA; b The Graduate School of Biomedical Sciences, Icahn School of Medicine at Mount Sinai, New York, New York, USA; c Roche Pharma Research and Early Development, Immunology, Inflammation and Infectious Diseases Discovery and Translational Area, Roche Innovation Center, New York, New York, USA; d Roche Pharma Research and Early Development, Immunology, Inflammation and Infectious Diseases Discovery and Translational Area, Roche Innovation Center, Basel, Switzerland; Boston University School of Medicine; University of Rochester; Emory University

**Keywords:** NS1 protein, RNA interference, influenza virus, systems biology, virus-host interactions

## Abstract

Viruses utilize a number of host factors in order to carry out their replication cycles. Influenza A virus (IAV) and human respiratory syncytial virus (RSV) both infect the tissues of the respiratory tract, and as such we hypothesize that they might require similar host factors. Several published genome-wide screens have identified putative IAV host factors; however, there is significant discordance between their hits. In order to build on this work, we integrated a variety of “OMICS” data sources using two complementary network analyses, yielding 51 genes enriched for both IAV and RSV replication. We designed a targeted small interfering RNA (siRNA)-based assay to screen these genes against IAV under robust conditions and identified 13 genes supported by two IAV subtypes in both primary and transformed human lung cells. One of these hits, RNA binding motif 14 (RBM14), was validated as a required host factor and furthermore was shown to relocalize to the nucleolus upon IAV infection but not with other viruses. Additionally, the IAV NS1 protein is both necessary and sufficient for RBM14 relocalization, and relocalization also requires the double-stranded RNA (dsRNA) binding capacity of NS1. This work reports the discovery of a new host requirement for IAV replication and exposes a novel example of interplay between IAV NS1 and the host protein, RBM14.

**IMPORTANCE** Influenza A virus (IAV) and respiratory syncytial virus (RSV) present major global disease burdens. There are high economic costs associated with morbidity as well as significant mortality rates, especially in developing countries, in children, and in the elderly. There are currently limited therapeutic options for these viruses, which underscores the need for novel research into virus biology that may lead to the discovery of new therapeutic approaches. This work extends existing research into host factors involved in virus replication and explores the interaction between IAV and one such host factor, RBM14. Further study to fully characterize this interaction may elucidate novel mechanisms used by the virus during its replication cycle and open new avenues for understanding virus biology.

## Abstract (precis)

Influenza A virus (IAV) and respiratory syncytial virus (RSV) present major global disease burdens. There are high economic costs associated with morbidity as well as significant mortality rates, especially in developing countries, in children, and in the elderly. There are currently limited therapeutic options for these viruses, which underscores the need for novel research into virus biology that may lead to the discovery of new therapeutic approaches. This work extends existing research into host factors involved in virus replication and explores the interaction between IAV and one such host factor, RBM14. Further study to fully characterize this interaction may elucidate novel mechanisms used by the virus during its replication cycle and open new avenues for understanding virus biology.

## INTRODUCTION

*Influenza A virus* (IAV) is a member of the family *Orthomyxoviridae* with a single-stranded, negative-sense RNA genome consisting of eight segments. IAV is responsible for an acute respiratory infection which affects 5% to 20% of the human population annually (1). While rates of mortality are not high in developed countries, the elderly and the young are at heightened risk and up to 650,000 people die annually due to influenza-related disease (http://www.who.int/news-room/detail/14-12-2017-up-to-650-000-people-die-of-respiratory-diseases-linked-to-seasonal-flu-each-year). Additionally, several pandemic strains with high virulence have emerged in the past and the threat of a novel pandemic strain is ever present. While IAV contributes considerably to population morbidity, human respiratory syncytial virus (RSV) represents a significant disease burden, specifically among children under 5 years of age and the elderly. RSV is a single-stranded, negative-sense RNA virus of the family *Pneumoviridae*, and it is the leading cause of hospitalization of children worldwide, accounting for three times more hospitalizations than IAV (2), and has a higher death rate than IAV among the elderly (3).

Viruses are obligate intracellular parasites, meaning that they rely on the cellular machinery of their hosts to execute their replication cycles. IAV and RSV both infect the tissues of the upper and lower respiratory tracts, specifically, the epithelial cells lining the airways (4). Due to these and other similarities, it is hypothesized that they may share a number of host factors required for their replication. To date, there have been multiple published reports of genome-wide screens uncovering host factors required for IAV replication (5–14). According to the data available at the time that our analysis was performed (5, 7–12), these published works identified 1,385 unique human genes required for IAV replication; however, there is significant discordance between the screen results. In fact, more than 93% of the hits were identified in only one screen. This lack of agreement is likely due to differing methodologies between the screens, such as the readout, cell type, virus strain, or reporter system used and, most importantly, the source of the small interfering RNA (siRNA) library. Further meta-analysis of these results does, however, reveal that, despite the disagreement on the gene level, there is significant overlap at the pathway level (15–17). These findings validate the overall RNA interference (RNAi) approach as a means to explore host factors required for virus replication; however, they also underscore the need for more-rigorous methodologies to discover and validate these host factors. To date, there have been no published, genome-wide RNAi screens identifying host factors involved in RSV replication.

In order to address the lack of agreement among the various IAV RNAi screens as well as to add novel information on host factors required for RSV replication, we employed an siRNA screen to explore host factors required for respiratory virus infection. We refined the available list of IAV host factors using an integrated “OMICS” methodology, incorporating data for both RSV and IAV, and integrated this using two complementary network analyses. This approach enabled the targeted screening of just 51 host factors with support for both IAV infection and RSV infection, 13 of which were validated with IAV.

We focused our efforts on one previously uncharacterized host factor, RNA binding motif 14 (RBM14). This protein was first identified in 2001 (18) and has been shown to modulate transcription and splicing of host genes in response to steroid hormone signaling (19), as well as to control DNA repair via interactions with the nonhomologous end joining (NHEJ) proteins (20). RBM14 is typically nuclear (21) and has been identified as a component of nuclear paraspeckles (22). Its known interactions with human viruses are limited to human immunodeficiency virus type 1 (HIV-1) (23, 24) and Epstein-Barr virus (EBV) (25). RBM14 has been implicated in innate immunity through its interaction with SMURF2 (26) and TRAF3 (27) and through its role in DNA sensing by cGAS (22). Here we report that RBM14 is required for efficient IAV replication and that it relocalizes to the nucleolus upon infection with IAV. The viral NS1 protein is both necessary and sufficient for this phenotype.

## RESULTS

### IAV and RSV elicit distinct gene expression profiles during infection.

**FIG 1:**
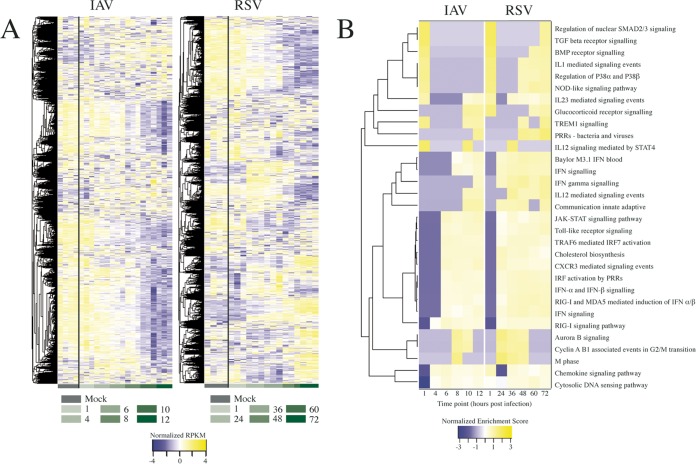
Host transcriptional responses following IAV or RSV infection. (A) Heat maps indicating the temporal expression profiles of all host genes in response to IAV or RSV infection of A549 cells as determined by mRNAseq analysis. Hierarchical clustering was performed to group genes based on similar expression profiles, indicated by the dendrograms to the left. Row-normalized RPKM values are shown in shades of color ranging from blue (downregulated) to yellow (upregulated). Time points are indicated below the panels in green shading. (B) Gene set enrichment was performed to identify pathways that are regulated during virus infection. The temporal enrichment scores for significantly positively regulated sets (*P* < 0.05) common to both IAV and RSV are shown here. Hierarchical clustering was performed, indicated by the dendrogram to the left. TGF, transforming growth factor; BMP, bone morphogenetic protein; PRR, pattern recognition receptor; IFN, interferon; IRF, interferon regulatory factor.

To complement and extend the publicly available array-based expression data, we performed mRNA sequencing (mRNAseq) analysis from A549 cells infected in parallel with influenza A/WSN/33 (H1N1) virus and RSV A/Long at various time points. RNA was extracted at the indicated times postinfection (PI), processed for mRNAseq analysis, and sequenced using an Illumina HiSeq 2000 system. The resulting sequences were mapped to human and viral transcripts, and transcript reads per kilobase per million (RPKM) values were calculated (see Table S1 in the supplemental material). This metric serves as a unit of transcript expression, normalizing for the length of each transcript. Monitoring temporal expression of viral gene transcripts confirmed increased viral gene expression indicative of viral replication (see Fig. S1A in the supplemental material). For analysis of host gene expression, RPKM values for each time point across both virus infections are represented as a heat map, with hierarchical clustering performed to group genes with similar temporal expression profiles (Fig. 1A). The results showed that IAV and RSV elicited distinct gene expression profiles in A549 cells, and principal-component analysis (PCA) demonstrated that these differences began at approximately 6 and 36 h postinfection for IAV and RSV, respectively (Fig. S1B), which mirrors the different kinetics of viral replication (Fig. S1A).

10.1128/mSphereDirect.00549-18.1 FIG S1 (A) A549 cells were infected with influenza A/WSN/33 (H1N1) virus or RSV A/Long at an MOI of 0.5 or 1.0, respectively. Cells were collected, and RNA was extracted at the indicated time points. Temporal profiles of all viral transcripts as determined by mRNAseq analysis are shown as numbers of reads per kilobase per million (RPKM) on the *y* axis. Error bars represent standard deviations. (B) Principal-component analysis of the mRNAseq data. The first 3 principal components accounted for 53.1% of the variance. PC1 accounted for 21.9% of the variance, PC2 for 18.1% of the variance, and PC3 for 13% of the variance. The mock-infected, IAV, and RSV samples diverge after the first four time points, indicated by a dashed red line. This indicates that at later time points, the two viruses elicit distinct gene expression profiles in A549 cells. Download FIG S1, TIF file, 1.5 MB. Copyright © 2018 Beyleveld et al. 2018 Beyleveld et al. This content is distributed under the terms of the Creative Commons Attribution 4.0 International license.

10.1128/mSphereDirect.00549-18.4 TABLE S1 The RPKM values from the mRNAseq data for each mapped gene and each viral time point are provided. The time point, virus, and two replicates (a and b) are indicated in the column header. Download Table S1, XLS file, 5.6 MB. Copyright © 2018 Beyleveld et al. 2018 Beyleveld et al. This content is distributed under the terms of the Creative Commons Attribution 4.0 International license.

In order to assess which biological pathways are affected by IAV and RSV infection, we performed gene set enrichment analysis (GSEA). GSEA shows whether predefined sets of genes show a statistically significant difference between samples—in this case, gene sets with defined biological functions were compared between infected and uninfected conditions in terms of their expression levels. In general, more gene sets are induced than suppressed by both viruses, and to identify significant pathways induced by both viruses, gene sets that achieved an adjusted *P* value of <0.05 for at least one time point were aggregated. A heat map of the enrichment scores for the 65 common and most significantly upregulated gene sets is shown in Fig. 1B. Many of these commonly enriched and upregulated pathways are related to the host response to virus infection and innate immune signaling pathways.

### Network analyses integrating multiple OMICS sources identify host factors likely to be involved in respiratory virus replication.

To screen for host factors required by IAV and RSV, we sought to employ a targeted siRNA-based screening assay. Gene targets were identified using network analyses which integrated our own mRNAseq profiling of IAV- and RSV-infected cells with multiple publicly available microarray profiling data sets, published genome-wide RNAi studies, virus-host and host-host protein interactome data (Table S2), and quantitative proteomics studies (Table S3). At the time that this study began, there were six published genome-wide RNAi screens (5, 7–11) identifying 1,291 unique host factors required for IAV replication (although there was little agreement between these screens on the gene level [15]) and there were (and still are) no genome-wide RNAi data available for RSV. This provided the motivation to perform an integrated analysis combining multiple data sources, with the goals of (i) identifying host factors with multiple levels of support for a role in virus replication and (ii) identifying host factors involved in both IAV and RSV life cycles.

10.1128/mSphereDirect.00549-18.5 TABLE S2 The host-host and virus-host protein-protein interaction data as curated from the literature are provided. For host-host interactions, the two interacting proteins are indicated on a single row. For virus-host interactions, the host protein is indicated alongside the virus protein with which it interacts. For the virus-host interaction data, the PubMed identifier (PMID) for each interaction is supplied. Download Table S2, XLS file, 5.0 MB. Copyright © 2018 Beyleveld et al. 2018 Beyleveld et al. This content is distributed under the terms of the Creative Commons Attribution 4.0 International license.

10.1128/mSphereDirect.00549-18.6 TABLE S3 The quantitative proteomics data for IAV and RSV are provided. The PMID for the source publication is indicated as a column header, and the presence of a protein in that publication is indicated. Download Table S3, XLS file, 0.7 MB. Copyright © 2018 Beyleveld et al. 2018 Beyleveld et al. This content is distributed under the terms of the Creative Commons Attribution 4.0 International license.

**FIG 2:**
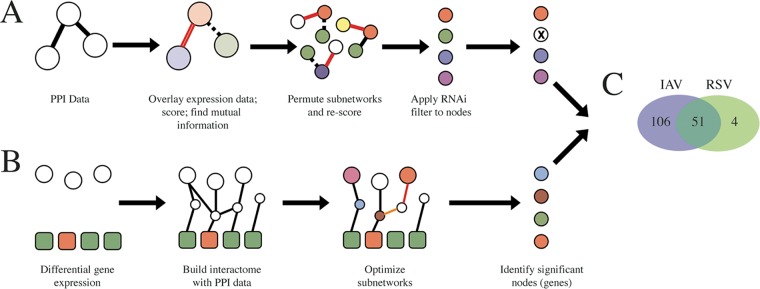
Schematic overview of the network analyses performed to generate the targeted list of host factors required for viral replication. (A) In the Mutual Information method, an interactome was assembled using protein-protein interaction data (empty circles), and gene expression data were overlaid (colored circles). Each subnetwork was scored by averaging the normalized expression values, and the discriminative potential of each subnetwork was determined based on the mutual information corresponding to its score and the class label of the expression data (i.e., infected versus mock). To identify significant subnetworks, their discriminative power was compared to that of randomly permuted subnetworks, retaining higher-scoring subnetworks. Finally, genes in significant subnetworks were filtered using the aggregate list of previously published RNAi hits from IAV (this step was not performed for RSV). (B) The SteinerNet method attempts to link RNAi screen hits (empty circles) from König et al. (10) to differential expression data (either mRNAseq or microarray data; colored squares) using a protein-protein interaction network. First, an interactome is assembled using PPI data. The subnetworks are mathematically optimized, and the genes in the optimal subnetworks are enriched for their requirement in viral replication. Due to the requirement for RNAi data, this analysis was only performed for influenza A virus. (C) The data from the two network analyses were aggregated, and results are shown in a Venn diagram. A total of 51 genes had shared support for IAV and RSV, while 106 and 4 had support for only IAV and only RSV, respectively.

Two complementary network analyses were performed. The Mutual Information method (28) (Fig. 2A) takes the protein-protein interaction (PPI) data and overlays expression data onto the subnetworks. Each subnetwork is scored by averaging its gene expression values, and a measure of the discriminative power of a subnetwork is determined by finding the mutual information between the subnetwork’s gene expression score and its class label (i.e., infected versus mock). Highly significant subnetworks are uncovered during an iterative permutation step, wherein nodes in the subnetworks are randomly replaced with first- and second-neighbors and the discriminative power is monitored for improvements. A list of enriched genes is derived from the elements in these highly significant networks, and the list is then filtered against the list of published siRNA hits, retaining only those that were previously identified in at least one siRNA screen. This analysis was performed for both IAV and RSV (except the final binary filter on RNAi hits, as these data are lacking for RSV) for both array-based expression data and our own mRNAseq data.

The SteinerNet method (28, 29) (Fig. 2B) requires phenotypic data as an input in the first step, and the raw IAV RNAi screen data from König et al. (2010) (9) were used for this purpose. Due to the lack of genome-wide RNAi data for RSV, this SteinerNet methodology was applied only to the IAV data sets. Initially, the phenotypic outcome data (i.e., the RNAi data) are physically linked to the gene expression profiling through a series of interactome subnetworks (PPI data). These subnetworks are subsequently mathematically optimized (using a prize-collecting variant of the Steiner tree problem [30]), wherein the subnetworks are scored for their complexity: adding too many genes or eliminating too many connections negatively weights a subnetwork; thus, the subnetworks with balanced complexity versus an ability to connect information are retained. As described above, the resulting optimal subnetworks consist of genes enriched for their requirement in viral replication.

The results from the Mutual Information and SteinerNet methods were aggregated, and a final list of 161 putative host factors was uncovered (Fig. 2C; see also Table S4), with 51 genes exhibiting shared support for both respiratory viruses in this study. Using an aggregate of the available quantitative proteomics data measuring protein abundance in response to IAV and RSV infection, a subset of 14 genes were found to pass all filters in this analysis (Fig. S2).

10.1128/mSphereDirect.00549-18.2 FIG S2 Venn diagram showing the results from the network analyses. Following the Mutual Information and SteinerNet network analyses, 157 and 55 genes were identified as putative host factors for influenza virus and RSV, respectively. Subsequent comparison to the available quantitative proteomics data revealed that 15 genes survived all category filters. One of these genes, TUBB (originally from the public array data), was not mapped in the mRNAseq data and was subsequently removed from the data set for all future analyses. Download FIG S2, TIF file, 0.2 MB. Copyright © 2018 Beyleveld et al. 2018 Beyleveld et al. This content is distributed under the terms of the Creative Commons Attribution 4.0 International license.

10.1128/mSphereDirect.00549-18.7 TABLE S4 A summary of the network analyses is provided. The total number of IAV siRNA screens in which a particular gene was identified is indicated in column C. Columns D and E, respectively, indicate whether the gene was identified by the network analyses for IAV and RSV. Columns F and G tally the number of quantitative proteomics studies that identified that particular protein. Column H is a sum of the previous columns for the purposes of sorting genes with higher levels of OMICS support, and column I indicates whether a gene was identified by both the IAV and RSV network analyses. Download Table S4, XLS file, 0.2 MB. Copyright © 2018 Beyleveld et al. 2018 Beyleveld et al. This content is distributed under the terms of the Creative Commons Attribution 4.0 International license.

### siRNA screening identifies previously characterized and novel host factors required by IAV.

**FIG 3:**
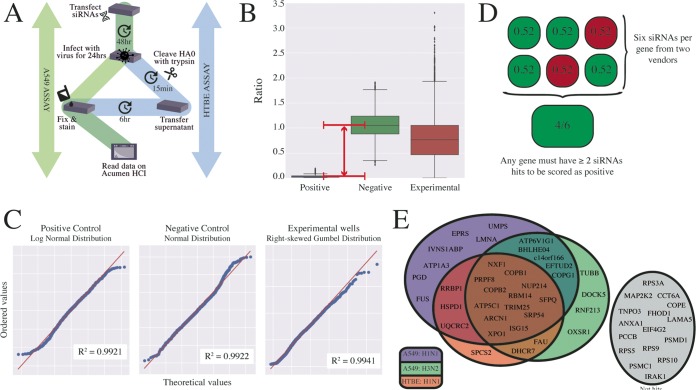
A targeted siRNA screen identifies IAV-required host factors. (A) Schematic representation of the assay used to screen putative host factors for their requirement in influenza virus replication. The green pathway (left) describes the A549 cell-based screen using influenza A/WSN/3 (H1N1) virus and influenza A/Pan/99 (H3N2) virus. The blue pathway (right) describes the follow-up screen performed in primary human tracheal bronchial epithelial (HTBE) cells. (B) Box-and-whisker plots showing the distribution of the raw values from the positive control and the negative control and the experimental data for the A549 cell-based screen (across both IAV subtypes), after the removal of outliers was performed using a Bayesian statistical approach. The first and third quartiles of the remaining data are indicated by the boundaries of the box, with the median indicated within the box. The whiskers represent the extreme boundaries of the data, with extreme values (values exceeding 1.5× the interquartile range beyond the median) plotted as points beyond the whiskers. The window between the positive and negative controls is indicated by a red arrow. (C) The raw data from the A549 cell-based screen were plotted against ideal probability distributions in quantile-quantile (QQ) plots (a graphical method for comparing probability distributions). If data from two distributions are similar, they lie approximately on the line *y* = *x* (red). Pearson correlation coefficients measure the degree to which the data fit the proposed probability distribution. The positive control (siRNA targeting influenza virus NP protein) follows a log-normal distribution (R2 = 0.9921), the negative control (nontargeting siRNA) follows a normal distribution (R2 = 0.9922), and the experimental data (siRNAs targeting putative host factors) follow a right-skewed Gumbel distribution (R2 = 0.9941). (D) Example data from an experimental condition are shown to illustrate the hit selection criteria. An siRNA targeting a candidate gene was required to reduce the normalized percentage of infection below 0.6 to be characterized as a hit (shown in green versus nonhits shown in red). Finally, for a gene to be scored as a hit in any screen it was required that at least two siRNAs for a gene candidate met these criteria. (E) The results from the two A549-based screens (purple and green) and the HTBE screen (red) are summarized in a Venn diagram. In the A549 cell-based screen, seven and four genes were uniquely identified in the H1N1 (purple) and H3N2 (green) variants, respectively. A total of 18 genes were identified in both A549 screens. These 18 genes (along with 8 genes from the pilot group) were additionally screened in HTBEs with influenza A/WSN/33 (H1N1) virus. Thirteen genes (brown section) were hits in all three variations of the screen. Gene candidates that were not scored as hits are shown in gray.

Using an siRNA screen assay (outlined in Fig. 3A), we performed a targeted RNAi screen of the 51 host factors predicted to be involved in the replication of both IAV and RSV. The screen was designed and optimized for replication of IAV in two respiratory cell types: human lung adenocarcinoma (A549) cells and primary human tracheal bronchial epithelial (HTBE) cells. A total of six siRNAs from two vendors were tested per gene to limit potential off-target effects. A nontargeting siRNA and an IAV NP-targeting siRNA served as a negative control and a positive control, respectively. Briefly, siRNAs were transfected into cells and incubated for 48 h to allow knockdown. Subsequently, cells were infected at a low multiplicity with either influenza A/WSN/33 (H1N1) virus (WSN) or influenza A/Panama/99 (H3N2) virus (Pan/99) for 24 h to achieve multicycle virus growth. In the case of the A549-based screen (see green shading in Fig. 3A), cells were fixed and immunostained for IAV NP protein. Undifferentiated HTBE cells lack the necessary proteases to cleave the hemagglutinin protein (and also do not tolerate exogenous trypsin well), rendering nascent virus noninfectious and thus preventing multicycle virus growth. To overcome this, supernatants were harvested from HTBE cells (see the blue shading in Fig. 3A), treated with exogenous trypsin, and then used to infect A549 cells for 6 h, followed by NP immunostaining as described above. After staining was performed, data were collected on an Acumen high-content imager (TTP Labtech). Box-and-whisker plots of the experiment-wide raw data, grouped by the controls and the experimental condition, are presented in Fig. 3B (see also Table S5). The positive control (siNP) achieves a median ratio of approximately zero, while the negative control (siNT) has a median ratio of approximately 1.0. A Bayesian statistics approach was used to identify and remove outliers. Briefly, the probability distributions of each data type (positive control, negative control, and experimental data) were confirmed by quantile-quantile (QQ) plot (Fig. 3C) and probability thresholds of 0.05 and 0.01 were used to exclude outliers from the control data and the experimental data, respectively. Using this approach, 5.44% of the positive-control data, 2.71% of the negative-control data and 0.19% of the experimental data were characterized as outliers and withheld from further analysis. Following the removal of outliers, the batch-specific means were calculated by Bayesian inference and the normalized percentage of inhibition was calculated for each well using these means. Strictly standardized mean difference (SSMD) values (31) were used to ensure data quality throughout the experiment. The experiment-wide SSMD value was 3.6, indicating a sufficiently large window between the positive and negative controls (given the observed error) to identify hits in this screen with confidence. In order to score an siRNA as a hit, we set the normalized percent inhibition (NPI) threshold at 0.6 (see green squares in Fig. 3D, top panel). For a gene to score as a hit, we required at least two of six siRNAs per gene to meet this threshold. All assays were performed with two or three technical repeats and two or three biological repeats per condition, leading to *n* = 4 to 9 for each siRNA in each screen. A pilot group consisting of the 14 genes which passed all OMICS filters (Fig. S2, center panel) was screened initially in the A549-based assay; subsequently, the remaining 37 genes with shared support for IAV and RSV were screened. The pilot group, as well as those genes that represented hits in either the A549-based WSN assays or Pan/99 assays, were additionally screened against WSN in HTBE cells. A Venn diagram of the results from the screens is shown in Fig. 3E (see also Table S5). Genes that were not hits in any of the screen variations are shown in gray to the right in the figure. The screen identified several host factors already known to play a role in IAV infection, including XPO1 (32), SFPQ (33), and the members of the COPI complex (34), which validates the strength of the screen. Of those hits that have not been previously implicated in IAV infection, we chose RBM14 for further characterization as it was a hit in all three variations of the screen.

10.1128/mSphereDirect.00549-18.8 TABLE S5 Results from the siRNA screen in A549 cells and HTBE cells. Column D specifies the siRNA for that gene, where “D” represents siRNAs from Dharmacon and “Q” represents siRNAs from Qiagen. Column E contains the final mean NPI value calculated for each siRNA, and column F indicates whether that siRNA was characterized as a hit. Download Table S5, XLS file, 0.1 MB. Copyright © 2018 Beyleveld et al. 2018 Beyleveld et al. This content is distributed under the terms of the Creative Commons Attribution 4.0 International license.

### Effect of RBM14 depletion on influenza virus replication.

**FIG 4:**
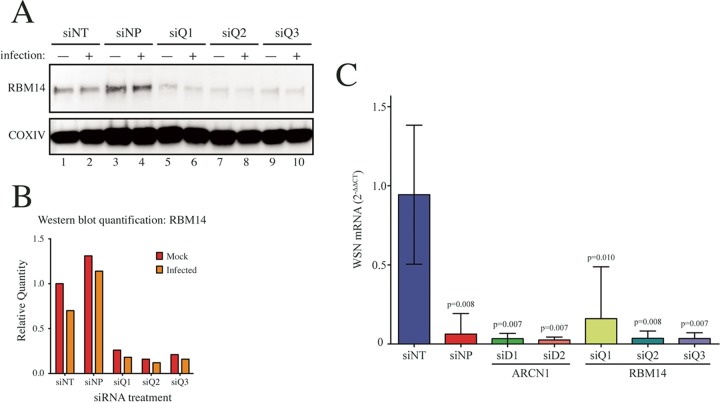
Validation of RBM14 as an IAV-required host factor. (A) A549 cells were transfected with three RBM14-targeting siRNAs (siQ1, siQ2, and siQ3). At 48 h posttransfection, the cells were infected with influenza A/WSN/33 (H1N1) virus at an MOI of 0.01 for 24 h. Protein lysates were prepared and subjected to SDS-PAGE and Western blot analysis, immunoblotting for RBM14 (top panel) and the loading control COXIV (bottom panel). (B) RBM14 levels were quantified from the Western blot shown in panel A. (C) A549 cells were transfected with three RBM14 siRNAs (siQ1, siQ2, and siQ3), two siRNAs targeting ARCN1 (siD1 and siD2), the positive-control siNP, or the negative-control siNT. At 48 h posttransfection, the cells were infected with influenza A/WSN/33 (H1N1) virus at an MOI of 0.01 for 24 h. Cellular RNA was extracted, and the expression level of WSN neuraminidase (NA) mRNA was measured by qRT-PCR and normalized to GAPDH in each sample and then to the siNT control. Error bars indicate standard deviations of the means. Data shown represent results of three technical repeats for each of three biological repeats. Statistically significant *P* values are shown in the figure.

To first validate the depletion of RBM14 by siRNAs, A549 cells were transfected with three siRNAs targeting RBM14 and were infected 48 h later with influenza A/WSN/33 (H1N1) virus. At 72 h posttransfection, cell lysates were collected and Western blot analysis showed that the RBM14 protein levels were efficiently depleted by all siRNAs (Fig. 4A and B). To monitor the effect of RBM14 depletion on virus replication, A549 cells were transfected with siRNAs and infected as described above, and then viral mRNA expression (NA segment) was measured by quantitative reverse transcription-PCR (qRT-PCR) at 24 h postinfection (Fig. 4C). Three biological replicates of each sample were included, and each was assayed in triplicate. In addition to the RBM14 siRNAs, two siRNAs targeting ARCN1 were included, as ARCN1 was another of our screen hits and has been previously validated as a required IAV host factor (34). WSN mRNA levels were normalized to GAPDH (glyceraldehyde-3-phosphate dehydrogenase) in each sample, and relative infection levels were normalized to the nontargeting siRNA (siNT). The positive control (siNP) reduced viral mRNA levels by ∼20-fold (*P* < 0.01). Similarly, siRNA-mediated depletion of ARCN1 and RBM14 reduced viral mRNA levels by ∼20-fold (*P* < 0.01) for all siRNAs except RBM14 siQ1, which showed ∼10-fold reduction (*P* < 0.05).

### RBM14 relocalizes to the nucleolus in response to influenza A infection.

**FIG 5:**
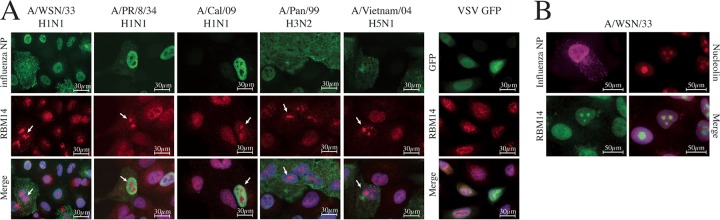
RBM14 relocalizes to the nucleolus in IAV-infected cells. (A) Wide-field immunofluorescence microscopy images show that RBM14 relocalizes to a distinct subnuclear compartment in IAV-infected cells. A549 cells were infected with the indicated influenza A viruses at an MOI of 1.0 and were fixed 6 h postinfection. The cells were stained with anti-RBM14 antibody (red), anti-IAV NP antibody (green), and DAPI nuclear stain (blue). RBM14 relocalization (indicated by white arrows) was observed with a range of influenza A virus subtypes but not with vesicular stomatitis virus (VSV; far right panel). Magnification, ×63. Scale bars are indicated on the figure. (B) WSN-infected A549 cells were stained with anti-nucleolin antibody (red), and it was observed that this staining coincided with anti-RBM14 staining (green) in infected cells (stained with anti-IAV NP antibody; magenta), indicating that IAV drives RBM14 to the nucleolus in infected cells. Magnification, ×63. Scale bars are indicated on the figure.

In order to explore the role of RBM14 in IAV infection, we investigated the subcellular localization of RBM14 in infected cells. A549 cells were infected with WSN at a multiplicity of infection (MOI) of 1.0 for 24 h, and the cells were then fixed and immunostained with anti-NP and anti-RBM14 antibodies for analysis by wide-field immunofluorescence microscopy (Fig. 5A, panel 1). A striking relocalization phenotype was observed in response to IAV infection. While RBM14 exhibits a diffuse nuclear staining pattern with some cytoplasmic signal in noninfected cells, it is clearly visible in distinct subnuclear compartments in virus-infected cells. Furthermore, we tested a variety of IAVs representing different subtypes (H1N1, H3N2, and H5N1), including both mouse-adapted strains and human viruses (Fig. 5A, panels 2 to 5), and found that the RBM14 relocalization phenotype was consistent across these influenza A viruses. In contrast, RBM14 does not relocalize in response to infection with vesicular stomatitis virus (VSV; Fig. 5A, far right panel), influenza B virus, Sendai virus, or RSV (data not shown). We additionally immunostained WSN-infected A549 cells with anti-nucleolin and found that the RBM14 signal coincided with the nucleolus in the infected cells (Fig. 5B). This phenotype was observed as early as 2 to 4 h postinfection and persisted throughout the course of the viral replication cycle (Fig. S3).

10.1128/mSphereDirect.00549-18.3 FIG S3 A549 cells were infected with influenza A/WSN/33 (H1N1) virus at an MOI of 1.0 for the times indicated before fixing and staining for immunofluorescence with anti-NP (green), anti-RBM14 (red), and DAPI were performed. Wide-field microscopy revealed that RBM14 relocalized to the nucleolus as early as 2 h postinfection (white arrows). Magnification, ×63. Scale bars are indicated on the figure. Download FIG S3, TIF file, 1.1 MB. Copyright © 2018 Beyleveld et al. 2018 Beyleveld et al. This content is distributed under the terms of the Creative Commons Attribution 4.0 International license.

### The role of influenza NS1 protein in RBM14 nucleolar relocalization.

**FIG 6:**
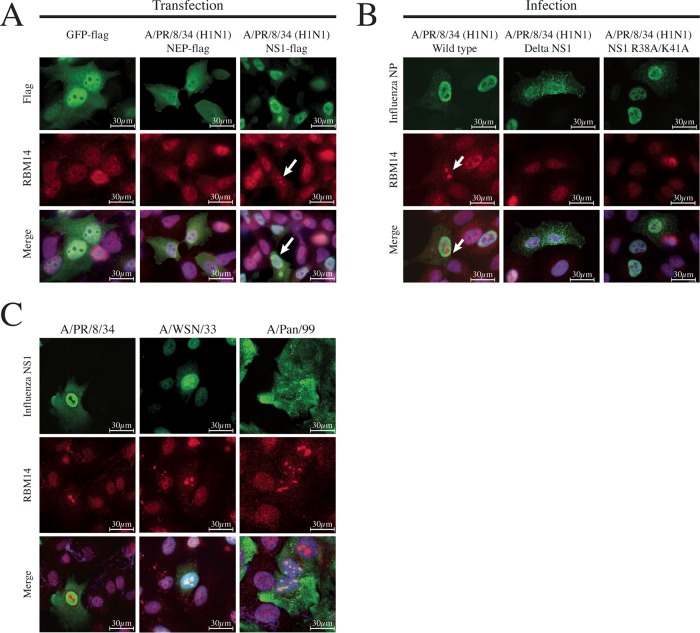
NS1 is necessary and sufficient for RBM14 nucleolar relocalization. (A) Individual Flag-tagged IAV expression plasmids were transfected into A549 cells and subsequently fixed and stained with anti-Flag antibody (green), anti-RBM14 antibody (red), and DAPI 24 h posttransfection. GFP-Flag and NEP-Flag (a representative IAV protein) did not elicit RBM14 relocalization (left and middle panels), while NS1-Flag did exhibit this relocalization phenotype (indicated by white arrow). Magnification, ×63. Scale bars are indicated on the figure. (B) A549 cells were infected with wild-type influenza A/PR/8 (H1N1) virus (left panel), influenza A/PR/8 (H1N1) ΔNS1 virus (middle panel), and recombinant PR/8 dsRNA binding NS1 mutant R38A/K41A (right panel), and cells were fixed 6 h postinfection. Cells were stained with anti-IAV NP antibody (green), anti-RBM14 antibody (red), and DAPI. By wide-field microscopy, RBM14 was observed to relocalize upon infection with wild-type virus (white arrow) but not with ΔNS1 virus or the dsRNA binding mutant NS1 virus. Magnification, ×63. Scale bars are indicated on the figure. (C) A549 cells were infected with three influenza A viruses: A/PR/8 (H1N1), A/WSN/33 (H1N1), and A/Pan/99 (H3N2). The cells were fixed 6 h postinfection and stained with anti-NS1 (green), anti-RBM14 (red), and DAPI. Wide-field microscopy shows that PR/8 NS1 (left panel) did not localize to the nucleolus at 6 h; however, the NS1 proteins from WSN and Pan/99 (middle and right panels) exhibited mild and strong nucleolar localization, respectively. Magnification, ×63. Scale bars are indicated on the figure.

In order to determine if a specific IAV protein was responsible for RBM14 relocalization, individual influenza A/PR/8/34 (H1N1) virus Flag-tagged protein expression plasmids were transfected into A549 cells. At 24 h posttransfection, the cells were fixed and immunostained with anti-RBM14 and anti-Flag antibodies and then imaged by wide-field immunofluorescence microscopy. Expression of the NS1 protein alone was sufficient to elicit RBM14 relocalization (Fig. 6A, right panel), while a control protein (Flag-tagged green fluorescent protein [GFP]; Fig. 6A, left panel) and the other viral proteins (nuclear export protein [NEP] is shown as a representative; Fig. 6A, middle panel) did not. To confirm the requirement for NS1 protein, A549 cells were infected with wild-type virus or ΔNS1 influenza A/PR/8/34 (H1N1) virus for 24 h and then fixed and immunostained for wide-field fluorescence microscopy as described above. Wild-type PR/8 virus induced a robust RBM14 relocalization phenotype (Fig. 6B, left panel), while this was starkly absent in the ΔNS1 PR/8-infected cells (Fig. 6B, middle panel). A double-stranded RNA (dsRNA) binding mutant NS1 virus (R38A/K41A) (35) also failed to bring about RBM14 relocalization to the nucleolus (Fig. 6B, right panel). Since it has been reported that the NS1 proteins of different IAV strains differentially localize to the nucleolus during infection, A549 cells were infected with PR/8, WSN, and Pan/99 viruses at an MOI of 1.0 for 24 h and the cells were fixed and immunostained with anti-RBM14 and anti-NS1 antibodies for imaging by wide-field immunofluorescence microscopy. While all of the viruses induced RBM14 nucleolar relocalization, they exhibited zero, medium, and high levels of NS1 nucleolar localization, respectively (Fig. 6C). Thus, there does not appear to be a direct link between RBM14 nucleolar relocalization and the presence of NS1 in the nucleolus.

## DISCUSSION

Since IAV and RSV infect similar biological niches and because all viruses rely on the host cell for basic replication functions (intracellular transport, genome replication, transcription and translation, etc.), it was hypothesized that they might share common host factors. Understanding which elements of the human proteome a virus relies upon for its replication cycle, and characterizing how these proteins are used by the virus, helps increase our understanding of virus biology. Furthermore, there is a potential to uncover novel drug targets that are independent of the virus itself, thus reducing the potential for the virus to develop resistance mutations and possibly uncovering drugs that work effectively on multiple viruses. While several genome-wide RNAi screens searching for host factors for IAV existed at the time that this study was commenced (5, 7–11), the aggregated hits from those screens had little overlap (15, 16). Gene ontological (GO) analysis of the combined results from some of these works revealed collective agreement at the level of functional pathways (15, 17) and suggested a promising foundation for a more targeted approach. To this end, OMICS data (expression profiling study results, existing RNAi screen results, host-host and host-virus interactomes, and quantitative proteomics) were gathered for both viruses (however, no RNAi studies were available for RSV) and were integrated by network analyses. To supplement the existing expression profiling data (from publicly available microarray-based expression experiments), we performed a temporal mRNAseq analysis of human cells infected in parallel with IAV and RSV. The mRNAseq analysis indicated that these two viruses elicit distinct gene expression profiles in A549 cells, although a GSEA of the results reveals that many host pathways are commonly upregulated by both viruses, lending credence to our hypothesis. Next, we used two complementary network analysis methodologies to integrate these mRNAseq data with the published OMICS data and refine the global list of RNAi hits. The mutual information method made use of the virus-host interactome and the accompanying expression data to assemble subnetworks and score their ability to distinguish infected cells from uninfected cells as a measure of their potential involvement in viral replication. Similarly, the SteinerNet method linked the results of an RNAi screen (using the raw phenotypic data from König et al. [9]) to expression data by way of the interactome. Combined, these methodologies yielded a list of 51 genes with shared support for their involvement in IAV and RSV replication. Several of these genes are part of functional complexes, for example, those encoding the COPI-related proteins (COPB2, COPB1, ARCN1, COPA, and COPG) which are involved in transport of endosomes and have been shown to be required for viral replication (5, 8–10, 12, 36–38). PRPF8, SFPQ, EFTUD, and FUS are all linked to mRNA splicing (39), and at least SFPQ has already been validated as an influenza virus host factor (33). We focused the screen on this subset of 51 genes for its potential to uncover a pan-respiratory virus host factor, with this study specifically screening against influenza A virus.

Genome-wide screens assess a large number of candidates and are necessary in the early stages of research into host factors; however, their relatively low statistical rigor on the hit level is unavoidable due to the high-throughput nature. We screened 2-orders-of-magnitude-fewer genes, enabling a more thorough approach; we used both a cell line and primary cells, a larger plate format, two virus strains, multiple siRNAs per gene, and multiple technical and biological repeats. Thirty-seven of the 51 genes screened were identified as potential host factors for either WSN or Pan/99 in our screen, with shared support for 18 genes. Rescreening these hits in primary cells further confirmed 13 genes as hits across all variations of the screen. Within this final hit list, five genes (encoding ARCN1, NXF1, COPB2, PRPF8, and COPB1) had originally been identified in two or more RNAi studies and three (SFPQ [33], XPO1 [9], and ARCN1 [34]) have been validated as being required for influenza virus replication. ISG15 is a well-characterized interferon-stimulated gene (ISG), so its presence as a host factor required for viral replication was initially puzzling; however, work by Speer et al. (2016) (40) revealed that ISG15 depletion leads to a sustained antiviral response in cells due to interferon dysregulation. The remainder of the hits in our screen (ATP5C1, ISG15, NUP214, RBM14, SRP54, and TRIM25) were previously identified in only one RNAi study, which underscores how the discordance between the existing RNAi studies does not contraindicate the method; rather, the methodology differences between those screens simply could not capture all of the results with equal likelihood. Furthermore, our results reinforce the notion that supplementing the available RNAi data with a range of OMICS sources enriches the list for targets required for viral replication.

We sought to characterize a hit from our results to better understand its role in viral replication. RNA binding motif protein 14 (RBM14) has not been previously characterized in the context of respiratory virus infection; however, it has been shown to interact with human immunodeficiency virus type 1 (HIV-1) Rev (23) and Tat (24) proteins and to modulate the expression of p24 (41). For influenza virus, affinity-purification mass spectrometry (AP-MS) studies have linked RBM14 to the components of the IAV polymerase complex, NEP, and NS1 (42), but no validation has been performed. RBM14 has also been shown to interact with a noncoding RNA called EBV-encoded RNA 2 (EBER2) (25). RBM14 has been implicated in the innate immune response to DNA viruses through the cGAS-STING-IRF3 pathway (22), and this function is dependent on the formation of a ribonuclear complex involving the long noncoding RNA NEAT1, as well as several other paraspeckle proteins. RBM14 has also been found to interact with TRAF3 in immunocomplexes that arise in response to dsDNA and dsRNA sensors, further suggesting a role in the innate immune response to viral infection.

RBM14 siRNAs were confirmed by Western blotting to efficiently deplete RBM14 in A549 cells, and it was shown that the absence of RBM14 results in ∼10-fold to ∼20-fold reductions in IAV mRNA expression. Additionally, the depletion by siRNAs of a previously reported host factor, ARCN1, was also shown to reduce viral mRNA to similar levels. These results employ a measurement of viral replication orthogonal from that used in the screen to confirm the effects of RBM14 depletion on viral replication, lending further support to the notion that RBM14 is a host factor required for efficient IAV replication.

RBM14 is primarily nuclear with diffuse cytoplasmic signal in uninfected cells; however, we observed a stark nucleolar relocalization phenotype in response to IAV infection. This phenotype was apparent from as early as 2 h postinfection and was highly robust. Furthermore, it was observed across several IAV subtypes, suggesting that it represents a pan-IAV response. However, it appears to be specific to IAV as RBM14 did not relocalize to the nucleolus in response to VSV, influenza B virus, Sendai virus, or RSV. Transfection of individual IAV proteins into A549 cells revealed that the presence of IAV NS1 was sufficient to bring about the relocalization of RBM14 in the absence of other viral proteins. Furthermore, infection with ΔNS1 PR/8 virus failed to elicit relocalization of RBM14. Thus, IAV NS1 is both necessary and sufficient for the relocalization of RBM14. A recombinant virus expressing a mutant NS1 protein deficient in dsRNA binding (R38A/K41A) also failed to elicit RBM14 relocalization, indicating that this phenotype is dependent on the dsRNA binding capacity of NS1. The NS1 proteins of WSN and Pan/99 localize to the nucleolus (to differing degrees) during infection and the NS1 protein of PR/8 does not—yet all of these viruses elicit RBM14 nucleolar relocalization. Thus, the ability to relocalize RBM14 appears to be independent of the presence of NS1 in the nucleolus. Since these images were all captured at 6 h postinfection, it is possible that these viruses exhibit differential kinetics with respect to their nucleolar localization of NS1; however, previous studies have also described differential NS1 nucleolar localization between at least WSN and Pan/99 (43). Taking the data together, it is suggested that NS1 is not directly responsible for taking RBM14 to the nucleolus and thus that additional factors, perhaps involving RNA or RNA binding proteins, may be involved. Given that RBM14 contains conserved RNA recognition motifs and that the dsRNA binding-deficient NS1 mutant fails to cause relocalization, we hypothesize that the observed RBM14 phenotype is dependent on interactions with viral or cellular RNAs. Indeed, RBM14 is known to interact with the long noncoding RNA (lncRNA) NEAT1 in the paraspeckle (44). NEAT1 is induced by IAV infection, and this relocates SFPQ to the paraspeckle, thereby eliminating its transcriptional repression of interleukin-8 (IL-8) expression (45) and thus activating an innate immune pathway in response to viral infection. If a similar mechanism involving NEAT1 were involved in the sensing of RNA viruses, IAV NS1 might be driving the sequestration of RBM14 away from the paraspeckle to blunt this response. Additionally, RBM14 is an indirect coactivator of gene expression (18, 46) and as such its effects on gene expression may be modulated either by the host (in response to viral infection) or by the virus itself in an effort to subvert antiviral responses or to rewire the response to support virus replication. Given the NS1-driven relocalization of RBM14 and the well-characterized role of NS1 in suppression of innate immune responses to infection, it is conceivable that IAV NS1 may modulate any potential antiviral transcriptional effects of RBM14.

## MATERIALS AND METHODS

### Cell culture.

All cells in culture (unless specifically indicated otherwise) were incubated in Dulbecco modified Eagle medium (DMEM) (Corning) with 10% fetal bovine serum (FBS) (BioTC; Laboratory Disposable Products, Inc.) and 1× penicillin streptomycin (Corning) at 37°C and 5% CO_2_. A549 and MDCK cells were obtained from the ATCC. HTBE primary cells were obtained from Lonza (donor number 7F3506; catalog no. CC-2541) and were cultured in bronchial epithelial cell growth medium (BEGM) (Lonza; catalog no. CC-3171) supplemented with a BEGM BulletKit (Lonza; catalog no. CC-3170) (complete BEGM), in collagen-coated culture ware, maintaining the cells in the undifferentiated form. For passaging of HTBE cells, the medium was aspirated and the cells were washed with phosphate-buffered saline (PBS). A 5-ml volume of trypsin was added, and the cells were incubated at 37°C and 5% CO_2_ for approximately 3 min. An equal volume of trypsin neutralizing solution (TNS) was added to the cells, and the cells were collected by centrifugation at ∼1,500 × *g* for 3 min. The supernatant was aspirated, and the cells were resuspended in complete BEGM for seeding or passaging.

### mRNAseq.

A next-generation sequencing (NGS) library was prepared for mRNAseq analysis using TruSeq RNA library prep kit v2 (Illumina; San Diego, CA) from 1 μg of total RNA. Following mRNA isolation and fragmentation, cDNA synthesis generates double-stranded cDNA and Illumina barcode adaptors are ligated for sequencing. The NGS libraries were quantified using an Agilent Bioanalyzer and quantitative PCR (qPCR). The samples were randomized and pooled in groups of six, and the content of each pool was sequenced in one lane of a HiSeq flow cell with a paired-end (PE) read length of 50 bp using TruSeq SBS kit v3 (Illumina; catalog no. FC-401) and a HiSeq 2000 sequencing system (Illumina). Sample demultiplexing was performed using CASAVA v1.8.2 software (Illumina). The samples were then analyzed through the use of an internal quality control pipeline to assess total passed filter read count, quality scores, PCR duplicate levels, GC content, and nucleotide distribution. Short-read data from mRNAseq experiments were separately mapped against viral and human transcripts (Ensembl release 60; November 2010) with Bowtie2 (47), prioritizing for concordant paired alignments. All alignments were mapped for viral transcripts, while only unique alignments were mapped for human transcripts. The resulting SAM files were processed with SAMtools (48) to yield RPKM values calculated for each sample as described by Mortazavi et al. (49). The Ensembl identifiers were converted to canonical gene symbols using Entrez gene identifier tables from NCBI (https://www.ncbi.nlm.nih.gov/gene). For genes with two or more mapped transcripts, the RPKM values were averaged. Hence, expression levels for genes were obtained by summarizing all reads mapping to a gene in a sample and computing the RPKM value per gene. Reads mapping to multiple viral transcripts were assigned to their respective transcripts proportionally to the expression level of a transcript as measured by unique reads. As an initial quality control step, we evaluated the read depth for each sample. The average read depth exceeded 20 million reads per sample, and one sample with an unexpectedly large read depth was removed. Additionally, principal-component analysis was performed using Partek Genomics (v6.6beta; Partek, St. Louis, MO), which guided the removal of one mock RSV sample, two 60-h RSV samples, and one 48-h RSV sample. The triplicate samples per time point were processed separately; however, stable variance across the triplicate meant that they could be treated as technical repeats during downstream analysis. The fold change values relative to mock samples were used for gene set enrichment analysis (http://software.broadinstitute.org/gsea/index.jsp) using a custom GMT file compiled from Reactome v38 (50) NCI Nature gene sets (http://www.ndexbio.org/#/user/301a91c6-a37b-11e4-bda0-000c29202374) and selected Ingenuity canonical pathways (Ingenuity, Redwood City, CA). Enrichment maps for positively and negatively enriched gene sets having a *P* value of <0.05 were created with Cytoscape v2.82 using the Enrichment Map v1.2 plugin.

### Network analysis.

The IAV RNAi hits from the six published genome-wide RNAi screens (5, 7–11) were aggregated, and raw data from the König et al. (2010) screen were obtained. The host interactome data were compiled from three PPI databases: IntAct (July 23, 2012 version; www.ebi.ac.uk/intact), BioGrid v3.1.90 (www.thebiogrid.org), and the Database of Interacting Proteins (DIP; Dec 26, 2011 version; http://dip.doe-mbi.ucla.edu/dip/Main.cgi) (see Table S2 in the supplemental material). The data from those databases were aggregated and filtered to exclude genetically defined interactions, for example, suppressive/synthetic genetic interactions defined by inequality (BioGrid) or genetic interference (DIP). The aggregated host-host PPI databases comprised 105,488 interactions from 13,862 unique genes. Influenza A virus-host interactions were curated manually from VirHostNet, and the RSV-host PPI data set was curated from selected publications (51–54) (Table S2). Quantitative proteomics data were manually curated from studies on IAV (55–59) and RSV (54, 60) (Table S3). An aggregated gene expression data set was compiled from several publicly available sources: accession no. GSE19392, primary human tracheal bronchial epithelial (HTBE) cells infected with influenza A/PR/8/34 (H1N1) virus (8); accession no. GSE32139, HTBE cells infected with influenza A/Udorn/72 (H3N2) virus or RSV (rgRSV244) (61); and accession no. GSE3397, human bronchial epithelial cells (BEAS-2B) infected with RSV/Long (62). The data from Shapira et al. (8) (accession no. GSE19392) were analyzed separately with two sets of time-matched controls (mock and trypsin), and the results were aggregated. The array data were also normalized separately using quantile normalization (63, 64) and the supervised normalization method (65). Two complementary network analyses were utilized to prioritize existing RNAi hits derived from published studies of influenza A virus-infected cells. The mutual information method (28) was implemented using a custom script in the R programming environment (http://www.cran.r-project.org/) and the Information-Theoretic Measures library (https://cran.r-project.org/web/packages/infotheo/). For time course experiments, the data for individual time points were analyzed separately and the results for all time points merged. Three permutation tests (of genes, samples, and subnetworks) were performed to filter the initial results (*P* value of <0.05 for 200 gene permutations, 10,000 sample permutations, and 200 subnetwork permutations). Finally, the resulting list of genes enriched for virus replication was passed through a binary filter selecting genes that were identified in at least one siRNA screen (this step was omitted for RSV due to a lack of RNAi data). The SteinerNet method (29, 66) was used to analyze influenza A virus infection expression data using the fold change expression values as described above, RNAi confidence score data (9), and the previously described PPI data. Similarly, the individual time points from the various expression profiling experiments were analyzed separately and the results aggregated. Due to the lack of genome-wide RNAi studies for RSV, network analysis for RSV was limited to the mutual information method.

### A549-based siRNA screen.

siRNAs were reverse transfected into cells as follows. A 3.6-μl volume of 1 μM siRNA was diluted in 26.4 μl of Opti-MEM. A 0.2-μl volume of RNAiMax was diluted in 29.8 μl Opti-MEM and incubated at room temperature for 5 min. The diluted siRNA and RNAiMax solutions were combined at a 1:1 ratio and incubated for 20 min. Finally, a 60-μl volume of cells was added at a concentration of 1 × 10^5^ cells/ml to DMEM (20% FBS, no penicillin/streptomycin). At 6 h posttransfection, the medium was replaced with Opti-MEM containing 0.5% bovine serum albumin (BSA). At 48 h posttransfection, the cells were infected with influenza A/WSN/33 (H1N1) virus or influenza A/Pan/99 (H3N2) virus at an MOI of 0.1. The virus was diluted in PBS–0.5% BSA, and 30 μl was added per well for 1 h. The virus was aspirated, the cells were washed once with 100 μl PBS, and 100 μl of postinfection (PI) medium (Opti-MEM with 0.5% BSA and 0.25 μg/ml tosylsulfonyl phenylalanyl chloromethyl ketone [TPCK] trypsin) was added. Cells were incubated for 24 h postinfection.

### HTBE-based siRNA screen.

siRNAs were reverse transfected similarly to the method described for the A549-based screen above with the following differences. To reduce the dilution factor of the siRNA and RNAiMax mix, the mix was lowered to a 20 μl total volume; 16 μl of Opti-MEM was mixed with 0.3 μl RNAiMax for 5 min at room temperature. Subsequently, 3.6 μl of 1 μM siRNA was added and incubated for 20 min at room temperature. HTBE cells were trypsinized as described above and diluted to 1 × 10^5^ cells/ml in complete BEGM; a 100-μl volume of cells was added atop the siRNA and RNAiMax solution (for a total volume of 120 μl). The cells were incubated for 24 h before a 110-μl volume of medium was removed and replaced with 90 μl of fresh complete BEGM. At 48 h posttransfection, HTBE cells were infected with influenza A/WSN/33 virus at an MOI of 0.5 as follows: a 90-μl volume of BEGM was removed, and a 30-μl volume of virus diluted in complete BEGM was added for 1 h. Next, 80 μl of complete BEGM was added and the cells were incubated for a further 24 h. To assess the amount of virus present in the supernatants, 95 μl was transferred to an empty 96-well plate and 5 μl of 100 μg/ml TPCK trypsin was added for 15 min at room temperature. Next, 2-fold serial dilutions of this supernatant were used to infect monolayers of A549 cells seeded 24 h prior in the 96-well format (3 × 10^4^ cells/well in DMEM) with a volume of 40 μl for 1 h. Finally, 60 μl of DMEM was added atop the A459 and supernatants, and the cells were incubated for 6 h.

### Fixing, staining, and imaging of siRNA assays.

Following incubation (24 h in the case of the A549-based assay and 6 h in the case of the HTBE supernatant transfers), the cells were fixed by the addition of 100 μl 8% formaldehyde directly into the media and incubation at 37°C for 15 min. Following aspiration and one wash with PBS, the cells were permeabilized with 0.1% Triton X-100–PBS for 15 min and blocked in 1% BSA–PBS for 1 h at room temperature. The cells were stained with anti-NP HT103 (made by the Mount Sinai Hybridoma Center Shared Research Facility) (1:2,000 dilution) for 1 h at room temperature, washed three times with 200 μl PBS, and stained with anti-mouse IgG Alexa Fluor 488 secondary antibody (Invitrogen; 1:1,000 dilution) and 1 μg/ml of ethidium bromide mixed with 1% BSA–PBS for 45 min at room temperature. The cells were washed three times with 200 μl of PBS and stored in 200 μl of PBS for analysis. The siRNA screen data were collected using an Acumen HCI (TTP Labtech) laser scanning cytometer, detecting Alexa Fluor 488 (green) and Ethidium bromide (red) signals in two channels. Data were recorded as the total area (in square micrometers) of signal observed within a well for each channel and are represented as a ratio of these two measurements.

### Data analysis of siRNA screen.

While conducting the siRNA screen, the strictly standardized mean difference (SSMD) value was used as a quality control measure on a plate-by-plate basis, using the positive and negative controls. The SSMD value was required to be greater than 3.0; otherwise, the plates were discarded and the experiments repeated. The equation for SSMD is shown below:

$$\text{SSMD}=\frac{{\text{μ}}_{\text{si}}\text{NP}-{\text{μ}}_{\text{si}}\text{DH2}}{\sqrt{{\text{σ}}_{\text{siNP}}^{2}+{\text{σ}}_{\text{siDH2}}^{2}}}$$

A Bayesian statistics approach was used to estimate the mean for the positive and negative controls on each plate. This approach provided a robust method for excluding outliers in a consistent manner across all experiments. Quantile-quantile (Q-Q) plots were used to confirm the distributions of the control data. Subsequently, using the data from the entire experiment to inform the prior probability distributions for the controls, each plate’s positive-control and negative-control mean was determined by Bayesian inference. Additionally, the experiment-wide prior distribution enables the automatic identification of outliers. The threshold was set to 0.05, indicating a 95% confidence that the value belongs in the probability distribution. For the experimental readings, a threshold of 0.01 was used since the data were inherently more varied and we sought to exclude fewer data points from these data. Following removal of outliers and the calculation of the plate means for the positive and negative controls, the final experiment-wide SSMD value was calculated as described above. The normalized percent inhibition (NPI) for each experimental well was calculated as follows:

$$\text{NPI}=\frac{{\text{μ}}_{\text{si}}\text{NP}-{\text{χ}}_{\text{gene}}}{{\text{μ}}_{\text{si}}\text{NP}-{\text{μ}}_{\text{si}}\text{DH2}}$$

To assess the statistical magnitude of the effect of each gene knockout, the SSMD for each gene was also calculated using the following formula (67):

$$\text{SSMD}=\frac{\Gamma \left(\frac{n-1}{2}\right)}{\Gamma \left(\frac{n-2}{2}\right)}\sqrt{\frac{2}{n-1}}(\frac{\overset{¯}{d_{i}}}{s_{i}})$$

where Γ represents the gamma function, *n* represents the number of samples for that gene, and *d_i_* and *s_i_* represent the average and standard deviations of the difference between that experimental value and the mean of the negative control values. An SSMD threshold of >1.4 and an NPI threshold of <0.6 were used to select hits in this screen.

### siRNA knockdown.

For all knockdown experiments distinct from the siRNA screen, the following reverse transfection protocol was implemented in a 24-well format. siRNAs were diluted to a working concentration of 1 μM, and a 6-μl volume was increased to 50 μl in Opti-MEM (Gibco). A 1-μl volume of Lipofectamine RNAiMAX transfection reagent (Thermo Fisher Scientific) was increased to 50 μl in Opti-MEM and incubated for 5 min at room temperature. These two solutions were combined 1:1 and incubated at room temperature for 20 min. The cells were trypsinized and resuspended to 6 × 10^4^ cells/ml in complete media (without penicillin/streptomycin), and 500 μl of cells was added to each well for a final siRNA concentration of 10 nM. This protocol was scaled accordingly for larger well formats.

### SDS-PAGE and Western blotting.

Attached cells were prepared for SDS-PAGE and Western blotting by washing once with PBS and addition of 2× Laemmli buffer with 10% β-mercaptoethanol directly over cells. Lysates were heated to 80°C for approximately 30 min. Samples were resolved on 4% to 20% Mini-Protean TGX precast protein gels (Bio-Rad) using SeeBlue Plus2 prestained protein standard (Invitrogen). Gels were transferred to a nitrocellulose membrane using a mixed-molecular-weight (MW) protocol on a Trans-Blot Turbo transfer system (Bio-Rad) according to the instructions of the manufacturer. Membranes were blocked with 5% BSA–PBS for 1 h at room temperature. Membranes were immunostained in 1% BSA–PBS overnight at 4°C, followed by three washes in PBS–0.05% Tween 20 (PBS-T). Images were captured using a ChemiDoc XRS+ system (Bio-Rad).

### qPCR.

Cellular RNA was extracted using an RNeasy minikit (Invitrogen) following the manufacturer’s directions. cDNA corresponding to the viral mRNA of segment 6 (NA) was synthesized using tagged primers, thereby adding an 18-to-20-nucleotide tag at the 5′ end as follows: CCAGATCGTTCGAGTCGT. Reverse transcription was performed with the tagged primer as described by Lanford et al. (1994) (68). An 8-μl mixture containing the approximately 200 ng of total RNA and 10 pmol of tagged primer was heated for 10 min at 65°C, chilled immediately on ice for 5 min, and then heated again for 5 min at 60°C. A 12-μl volume of preheated reaction mixture (10 μl First Strand buffer [Invitrogen; 2×] and 2 μl Superscript III reverse transcriptase) was added and incubated for 1 h.

Quantitative PCR (qPCR) was performed with a SYBR green qPCR kit (Roche; catalog no. 507203180) using a LightCycler 480 system (Roche). A 3-μl volume of a 10-fold dilution of the cDNA was added to the qPCR reaction mixture (5 μl SYBR green qPCR mix, 1 μl of 2 μM forward primer, and 1 μl of 2 μM reverse primer). The qPCR cycles were as follows: 95°C for 10 min, followed by 40 cycles of 95°C for 15 s and 60°C for 1 min. The primers used were as follows: mRNAtag (reverse), 5′-CCAGATCGTTCGAGTCGT-3′; WSNseg6_1314F (forward), 5′-TGAATAGTGATACTGTAGATTGGTCT-3′.

### Immunofluorescence microscopy.

Cells for immunofluorescence were grown on 10-mm-diameter glass slides in tissue culture. Slides were fixed with 4% formaldehyde for 10 min, permeabilized in 1% Triton X-100–PBS for 10 min, and blocked in 1% BSA–PBS for 1 h at room temperature. Immunostaining was performed in 1% BSA–PBS for 1 h at room temperature, followed by three washes in PBS-T. Cells were counterstained with DAPI (4′,6-diamidino-2-phenylindole) by addition at a 1:3,000 dilution from a 1 mg/ml stock during the final wash step. Images were captured using an AxioImager Z2M widefield fluorescence microscope (Zeiss) or a LSM880 laser scanning confocal microscope (Zeiss). Images were processed using ZEN imaging software from Zeiss.

### Measuring viral replication.

Supernatants containing influenza A virus were diluted in a 10-fold serial dilution series as appropriate, and a 200-μl volume of each of six successive dilutions was used to infect a monolayer of MDCK cells in 6-well cell culture plates. Following infection, wells were washed once with PBS and overlaid with a 1:1 mixture of 2× Leibovitz’s L-15 medium (Thermo Fisher Scientific) and 2% agarose with 1 µg/ml TPCK-treated trypsin (Thermo Fisher Scientific). Overlays were allowed to harden at room temperature, and the plates were incubated at 37°C and 5% CO_2_ for 48 h. Monolayers were fixed and stained with 0.5% crystal violet solution–20% methanol. Overlays were removed, and plates were allowed to dry. Wells with more than 20 but fewer than 200 plaques were counted, and the final PFU count per milliliter was calculated using the following formula (where *n* represents the number of plaques):

$$\text{PFU/ml}=\frac{n}{0.2}\;\times \;\text{dilution factor}$$

A rapid immunofocus assay was also used to determine virus titers. Two-fold serial dilutions of virus supernatants were made down the columns of a 96-well tissue culture plate. MDCK cells had been seeded at 5,000 cells/well in a 96-well tissue culture plate 24 h prior. A 30-µl volume of each dilution was used to infect the MDCK cells for 1 h at 37°C and 5% CO_2_, the supernatant was aspirated, and the cells were washed once with PBS. A 100-µl volume of DMEM was added to each well, and the plates were incubated for 6 h. The cells were fixed with the addition of 100 µl 8% formaldehyde for 10 min. Following one wash with PBS, the cells were permeabilized with a 100-µl volume of 1% Triton X-100 for 10 min and then blocked in 1% BSA for 1 h at room temperature. The cells were stained for influenza A virus NP protein using anti-NP antibody (HT103) for 1 h and washed three times with PBS. The cells were stained with goat anti-mouse IgG Alexa Fluor 488-conjugated secondary antibody (Thermo Fisher Scientific) for 45 min and washed three times with PBS. Cells were counterstained with DAPI by addition at a 1:3,000 dilution from a 1 mg/ml stock during the final wash step. Infected cells were counted versus uninfected cells using a Celígo imaging cytometer (Nexcelom Biosciences). The number of infected cells from a well within the linear range of the dilution series was used to calculate the number of infectious units using the same equation as that described above.

### siRNAs and antibodies.

The IAV NP-targeting siRNA (5′-GGATCTTATTTCTTCGGAGTT -3′; 5′-TTCCTAGAATAAAGAAGCCTC -3′) was synthesized by Dharmacon GE Lifesciences. The nontargeting siRNA was siGENOME nontargeting siRNA no. 2 from Dharmacon. The siRNA sequences and sources for all siRNAs used in screening experiments are supplied in Table S6. The antibodies used against RBM14 were from Abcam (catalog no. AB70636; Western blotting at 1:2,000 dilution) and Genetex (catalog no. GTX112293; immunofluorescence at 1:300). The anti-influenza A virus NP antibody (HT103) and NS1 antibody (1A7) were made at the Mount Sinai Hybridoma Center Shared Research Facility. The anti-nucleolin antibody was from Invitrogen (catalog no. 39-6400). The anti-COXIV antibody was from Li-Cor (catalog no. 926-42214).

10.1128/mSphereDirect.00549-18.9 TABLE S6 siRNA sequences for all siRNAs used in RNAi screening. Columns A and B list the Entrez gene ID and gene symbol, respectively. Column C lists the designation given to each siRNA internally to distinguish between multiple siRNAs from each vendor. Columns D and E list the vendors and the catalog numbers for the siRNA. Column F lists the sense sequences of the siRNA. Download Table S6, XLS file, 0.1 MB. Copyright © 2018 Beyleveld et al. 2018 Beyleveld et al. This content is distributed under the terms of the Creative Commons Attribution 4.0 International license.

### Software packages.

Data visualizations were performed using GraphPad Prism (version 6.0 h for Mac; GraphPad Software, La Jolla, CA, USA).
