# Supplementary figures and images for: Nucleolar Relocalization of RBM14 by Influenza A Virus NS1 Protein
Source: mSphere. 2018 Nov 14;3(6):e00549-18. doi: 10.1128/mSphereDirect.00549-18 (PMC6236804; doi:10.1128/mSphereDirect.00549-18)

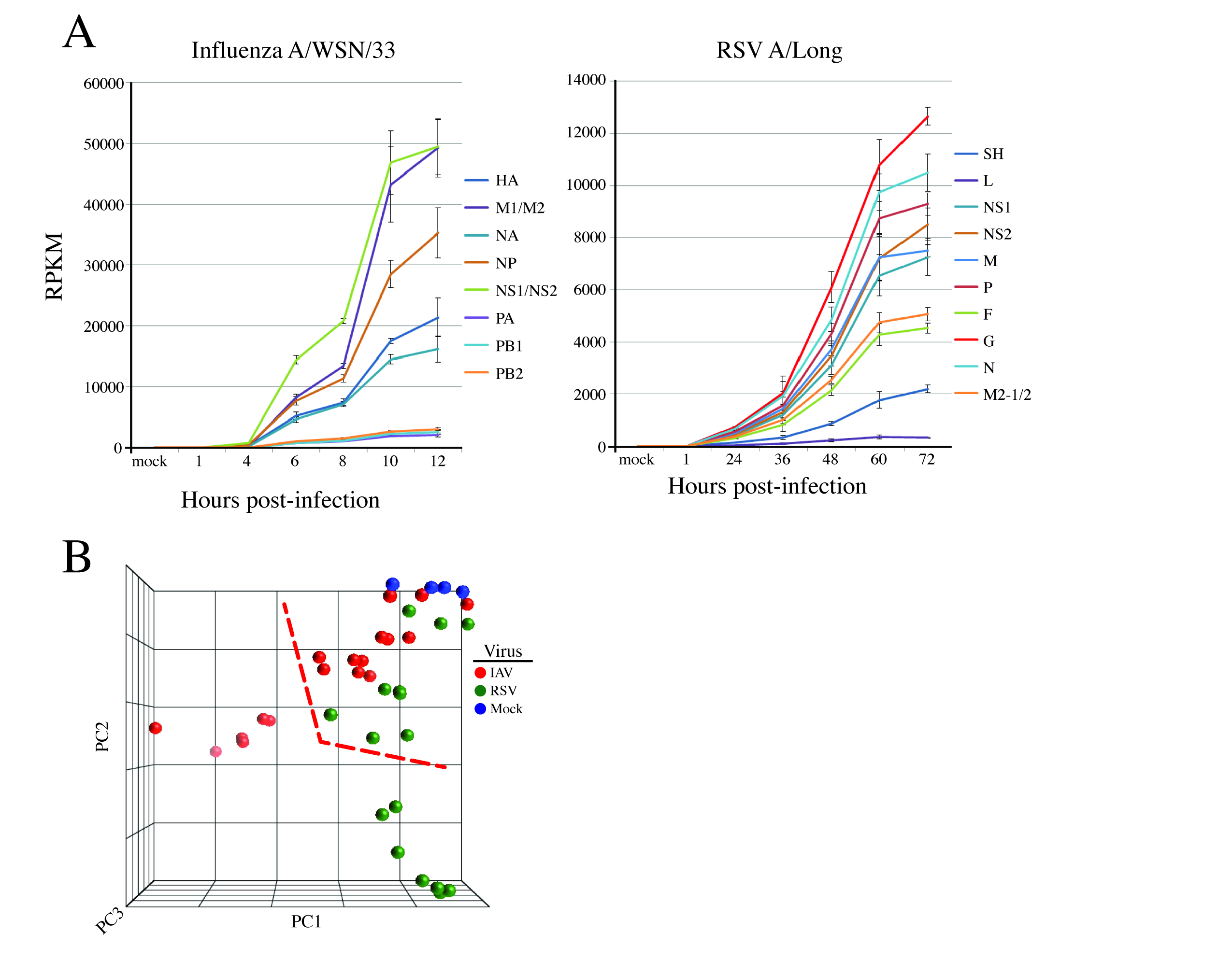

Supplement: FIG S1 [file sph006182706sf1.tif]

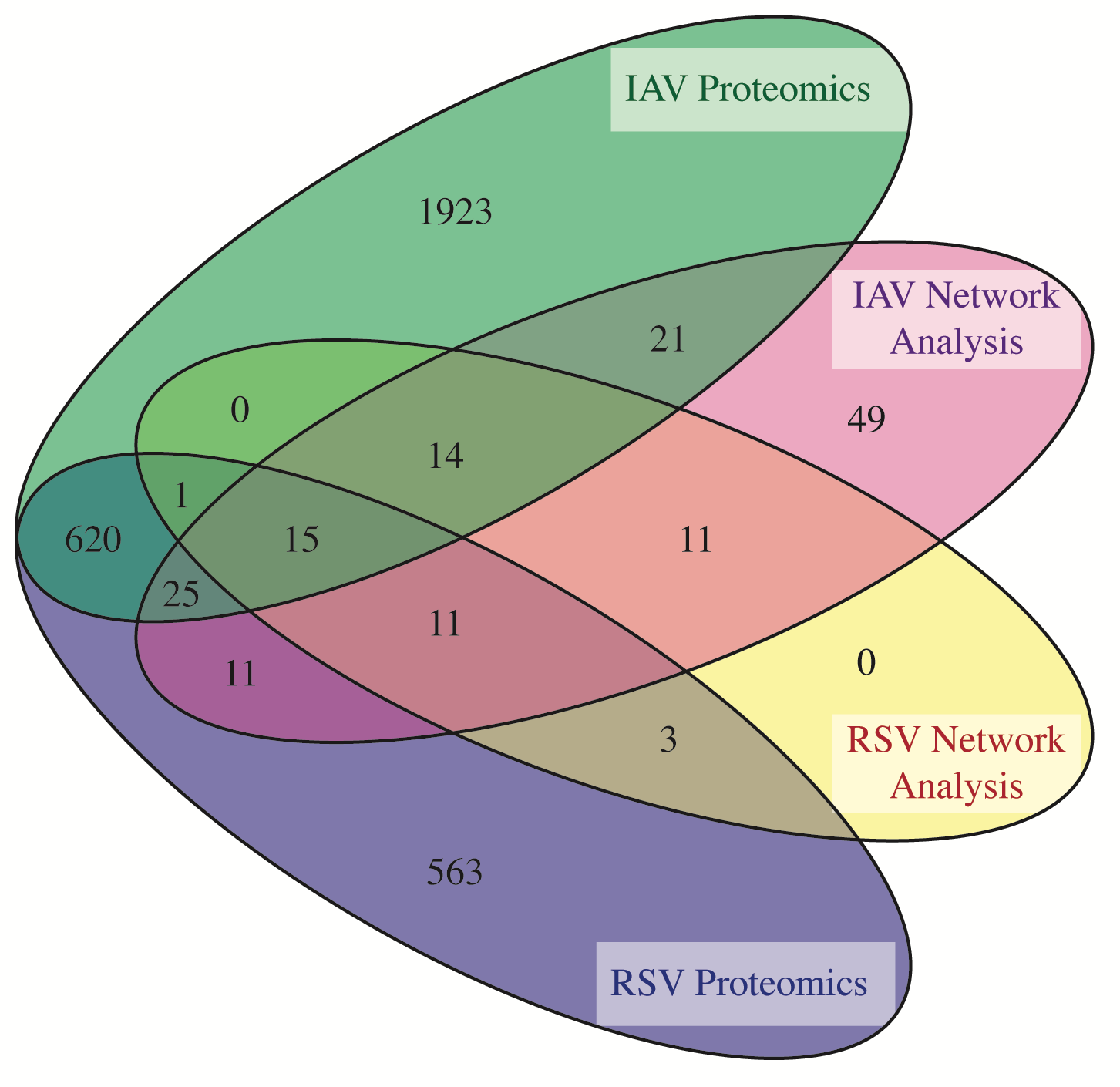

Supplement: FIG S2 [file sph006182706sf2.tif]

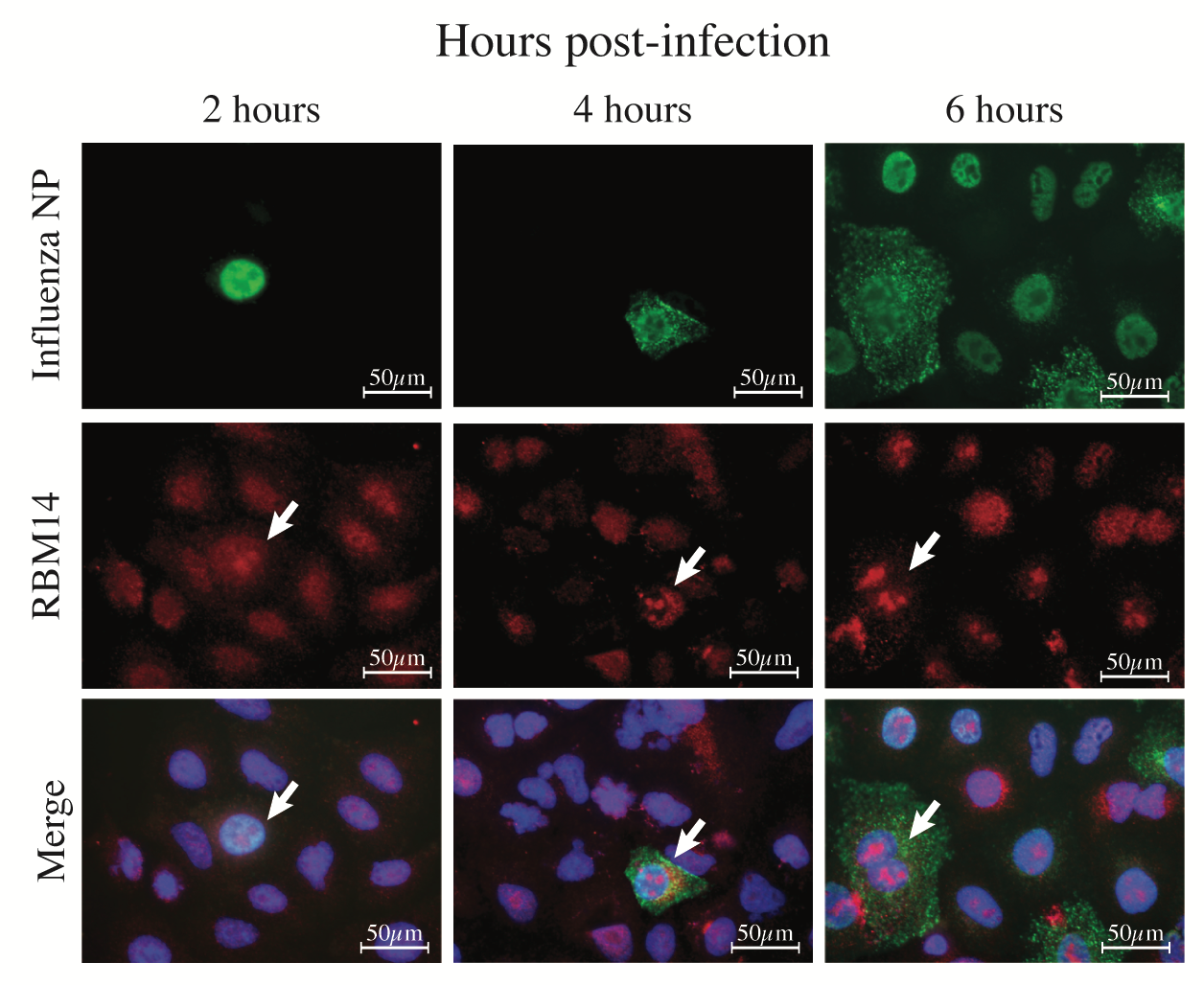

Supplement: FIG S3 [file sph006182706sf3.tif]
